# A randomised, placebo-controlled, triple-blind clinical trial to investigate the efficacy of *Ginkgo biloba* extract EGb 761^®^ in cognitive impairment associated with post COVID-19 syndrome—the EGb COCOS protocol

**DOI:** 10.3389/fnhum.2026.1658342

**Published:** 2026-06-15

**Authors:** Jordi A. Matias-Guiu, Katrin Arelin, Paula Jiménez Serrano, Anna Wacker, Marília Grando Sória, Martin Burkart, Udo Zifko

**Affiliations:** 1 Department of Neurology, Hospital Clínico San Carlos, Instituto de Investigación Sanitaria San Carlos (IdISSC), Universidad Complutense de Madrid, Madrid, Spain; 2 AES Part of Thermo Fisher Scientific, Leipzig, Germany; 3 MEDIAN Ambulantes Gesundheitszentrum, Leipzig, Germany; 4 Thermo Fisher Scientific, Wilmington, NC, United States; 5 Clinical Research, Dr. Willmar Schwabe GmbH & Co. KG, Karlsruhe, Germany; 6 Global Medical Affairs, Dr. Schwabe Holding SE & Co. KG, Karlsruhe, Germany; 7 Rudolfinerhaus Private Hospital GmbH, Vienna, Austria

**Keywords:** cognitive impairment, EGb 761®, long-COVID syndrome, post-acute Covid, randomised controlled trial, SARS-CoV-2, treatment

## Abstract

**Background:**

Cognitive impairment is frequent in post-COVID-19 syndrome (PCS). The understanding of the pathogenesis is still limited. Key factors such as neuroinflammation, neurovascular dysfunction, and disruption of cellular energy metabolism have been identified. There are no evidence-based treatments targeting the pathologic mechanisms of cognitive impairment associated with PCS available to date. Thus, treatment is directed towards symptom relief. EGb 761^®^, a dry extract from the leaves of *Ginkgo biloba* has anti-neuroinflammatory properties, improves microcirculation and neuronal mitochondrial function. Clinical efficacy in the treatment of cognitive impairment has been demonstrated. It is therefore reasonable to assume that the extract might be beneficial for use in cognitive impairment associated with PCS. Case series of patients with PCS reported significant improvement in cognitive function within 6 months of treatment. The EGb 761^®^ Post COVID Cognitive Impairment Study (EGb COCOS) aims to establish whether EGb 761^®^ is an effective treatment for cognitive impairment in PCS.

**Methods:**

In this prospective, multicentre, randomised, placebo-controlled, triple-blind trial, treatment effects and safety of EGb 761^®^ in patients with cognitive impairment associated with PCS will be investigated. Eligible patients aged ≥18 years with a history of probable or confirmed SARS-CoV-2 infection, diagnosis of PCS with cognitive symptoms that have been present for at least 2 months, objective cognitive impairment, and mild-to-moderate anxiety or depressive symptoms will be enrolled. Participants (*n* = 400 planned) will be randomised to oral, 12-week treatment with EGb 761^®^ (240 mg) or matching placebo once daily. The effect of EGb 761^®^ will be assessed on cognitive, neuropsychiatric, neurosensory, and functional outcomes. The analysis will be exploratory in nature, since generally accepted and validated primary endpoints have not been established. For safety, the incidence of adverse events (AEs) and serious AEs will be recorded.

**Discussion:**

The results of this trial will show for the first time whether EGb 761^®^ is an effective treatment for cognitive impairment in PCS.

**Clinical trial registration:**

https://euclinicaltrials.eu/ctis-public/view/2024-517199-39-00?lang=en, Identifier CTIS2024-517199-39-00.

## 1 Introduction

Post-COVID-19 syndrome (PCS, also referred to as long COVID) is defined as the absence of complete recovery after an acute COVID-19 episode caused by severe acute respiratory syndrome coronavirus 2 (SARS-CoV-2) infection (World Health Organization, 2021). According to the World Health Organization (WHO), PCS occurs in individuals with a history of probable or confirmed SARS-CoV-2 infection continuing at least beyond 3 months of the onset of COVID-19, with symptoms lasting at least 2 months, that cannot be explained by an alternative diagnosis (World Health Organization, 2021). However, there is no universally accepted definition of PCS to date (Sykes et al., 2021). PCS is associated with a wide range of overlapping symptoms. These include fatigue, muscle pain, shortness of breath, and cognitive dysfunction, which may fluctuate or relapse over time (GBD 2019 Mental Disorders Collaborators, 2022). The symptoms of PCS may be new-onset following initial recovery from the acute COVID-19 episode or persist from the original infection (World Health Organization, 2021). The prevalence of PCS is difficult to characterise (Soriano et al., 2022). It is estimated at 10% of non-hospitalised (Siso-Almirall et al., 2021) and approximately 85% of hospitalised COVID-19 cases (Carfi et al., 2020). The onset of PCS predominantly occurs in patients following moderate-to-severe infection. However, it may also be observed in patients who experienced mild or even asymptomatic infections (Perumal et al., 2023).

More than one-fifth of patients with PCS report cognitive impairment (Ceban et al., 2022). Attention, memory or executive functioning deficits are most prominent (Zifko et al., 2024). Neurocognitive symptoms have been documented for up to a year following acute infection and may persist longer. PCS with cognitive impairment has a substantial negative impact on daily functioning, contributing to a decrease in quality of life (Seessle et al., 2022) and productivity at work. It may lead to significant economic consequences for individuals and society (Rajan et al., 2021).

The pathophysiology of PCS-associated cognitive impairment is not fully understood. Key factors such as neuroinflammation, neurovascular dysfunction, and disruption of cellular energy metabolism have been identified (Monje and Iwasaki, 2022; Yang et al., 2021). Neuroimaging studies support the existence of structural and functional brain changes linked to abnormal cognitive performance (Chen et al., 2023; Diez-Cirarda et al., 2023). There are no evidence-based treatments that target the pathologic mechanisms of cognitive impairment associated with PCS. Thus, current management is directed towards symptom relief. Recommendations for cognitive impairment in this syndrome are based on assumptions, expert opinions, and experience from other disorders (Koczulla et al., 2022).

EGb 761®, a proprietary extract of Dr. Willmar Schwabe GmbH & Co. KG, Karlsruhe, Germany, is a dry extract from the leaves of *Ginkgo biloba* (maidenhair tree). It has shown benefits in improving cognitive impairment. Pharmacological data show reduction in blood viscosity, vasodilating effects, improvement of cerebral perfusion, as well as neuroprotective and anti-neuroinflammatory effects in animal models (Morató et al., 2024; Zifko et al., 2024). Standardized herbal extracts such as EGb 761® exhibiting a variety of pharmacological properties have been suggested to fit the treatment requirements for PCS caused by a complex pathophysiology. Specifically, the anti-oxidative and anti-inflammatory properties, improvements of mitochondrial function and improvement of neuroplasticity demonstrated for EGb 761® have been considered attractive for the treatment of cognitive impairment in PCS (Mueller and Müller, 2024). Moreover, EGb 761® protects endothelial cells, reduces blood viscosity, and improves microcirculation thereby targeting neurovascular dysfunction in PCS (Zifko et al., 2024). In preclinical models the compound has been shown to modulate neurotransmitters in the frontal cortex (Fehske et al., 2009, Yoshitake et al., 2010). Improved cognitive flexibility without changes in brain activation compatible with mild enhancement of prefrontal dopamine has been observed in a placebo-controlled trial (Beck et al., 2016). Case series of patients with PCS reported significant improvement in cognitive function and symptoms, such as fatigue and hyposmia, within 3 months of EGb 761® treatment (Gebetsroither, 2024; Zifko et al., 2022). The authors considered circulation-enhancing, antioxidant, and neuroplasticity effects of EGb 761® as plausible mechanisms of action and recommended randomised controlled clinical trials to confirm efficacy. Thus, the present randomised, placebo-controlled, triple-blind, clinical trial is warranted to further investigate efficacy and safety of EGb 761® as a promising therapy for patients with cognitive impairment associated with PCS.

## 2 Methods and analysis

### 2.1 Study design

EGb COCOS is a prospective, multicentre, parallel-group, randomised, placebo-controlled, triple-blind (participants, clinicians, and data analysts) clinical trial. It aims to address the current lack of established treatment for cognitive impairment in PCS and to contribute to the understanding of the potential role of EGb 761®. The trial will be conducted at multiple sites in Germany, Poland, and Spain. The trial sites will be a mix of hospital and specialised research centres.

### 2.2 Eligibility criteria

Key eligibility criteria are presented in Table 1. Each participant must meet all the inclusion criteria to be included; participants who meet any of the exclusion criteria will not be eligible for enrolment. Evaluation of eligibility criteria by investigators is based on thorough medical history taking, cognitive testing and questionnaires. The current protocol defines objective cognitive impairment as memory or executive functioning deficits. Memory deficits are assessed using the California Verbal Learning Test (CVLT), long delay-free recall, below the 50th percentile for age and education. Executive functioning deficits are assessed using the Trail-Making Test part B (TMT-B), below the 50th percentile for age and education.

**Table 1:** Specific inclusion and exclusion criteria.

Key inclusion criteria	Key exclusion criteria
Male or female outpatient, aged ≥18 years Has a diagnosis of PCS according to the WHO definition (World Health Organization, 2021) History of probable or confirmed SARS-CoV-2 infection, based on >1 of the following: positive PCR test at the time of infection positive antigen test at the time of infection along with clinical or epidemiological criteria physician’s diagnosis based on a positive PCR or antigen test and clinical or epidemiological criteria presence of immunoglobulin G-antibody to the viral nucleocapsid antigen (anti-N IgG), or presence of anti-S IgG antibodies in unvaccinated participants. Has persisting subjective cognitive problems for at least 2 months associated with PCS and arising after the SARS-CoV-2 infection. Has an objective cognitive impairment, defined as deficits in at least one of the following 2 domains of cognition: memory: CVLT, long delay-free recall, below 50th percentile for age and education executive functioning: TMT-B below 50th percentile for age and education. Has concomitant mild to moderate anxiety or depressive symptoms, defined as a GAD-7 score 5–14 and/or a PHQ-9 score 5–19.	Was on mechanical ventilation in an ICU during acute SARS-CoV-2 infection. Presents with haemorrhagic diatheses, coagulation disorder, gastric or duodenal ulcer. Has had acute or chronic neurologic diseases within the past 12 months (e.g., stroke, transient ischemic attack) or cognitive impairment before acute COVID-19. Has had acute or chronic psychiatric diseases within the past 12 months, including severe depression (PHQ-9 ≥ 20), severe anxiety (GAD-7 ≥ 15), significant primary sleep disorder, ADHD, bipolar disorder, substance use disorders, addictive behaviours, or schizophrenia. Mild to moderate psychiatric symptoms triggered by the SARS-CoV-2 infection will be allowed. Has a known hypersensitivity to one of the ingredients of the investigational product. Has taken *Ginkgo biloba* products within the past 12 weeks. Treatment with anticoagulants (eg, heparin, vitamin-K antagonists, dabigatran, edoxaban, apixaban, rivaroxaban) History of PEM persisting a week or longer in response to an exertion comparable to the planned site visits within the last 8 weeks

ADHD, attention deficit hyperactivity disorder; Anti-N IgG, immunoglobulin G-antibody to the viral nucleocapsid antigen; Anti-S IgG, immunoglobulin G-antibody to the viral spike antigen; CGI-I, Clinical Global Impressions-Improvement; CGI-S, Clinical Global Impressions-Severity; CVLT, California Verbal Learning Test; d2-R, GAD-7, generalised anxiety disorder; ICU, intensive care unit; IgG, immunoglobulin G; PCS, Post COVID-19 syndrome; PHQ-9, patient health questionnaire-9; PEM, postexertional malaise; SARS-CoV-2, severe acute respiratory syndrome coronavirus 2; SBQ-LC, Symptom Burden Questionnaire for long COVID; TMT-B, trail making test part B.

### 2.3 Recruitment

Patients will be recruited from the patient databases of the investigational sites, referrals from healthcare professionals, traditional paper advertising such as flyers and recruitment brochures, and via electronic methods, including social media advertising, keyword search engine optimisation and an email campaign.

### 2.4 Randomization, treatment allocation

This is a triple-blind trial with all participants, site personnel, and data analysts blinded to treatment. All members of the clinical trial team, including investigators, site staff, outcome assessors, and data analysts, will be blinded to treatment assignments whilst the trial is in progress. Interventions will be supplied in identical packages and the tablets will be similar in colour, smell, taste, and appearance.

Participants will be randomised using automatic interactive response technology (IRT) by allocating multiple complete blocks to each site. Each participant number will be associated with treatment arms according to a random process.

**Figure 1:**
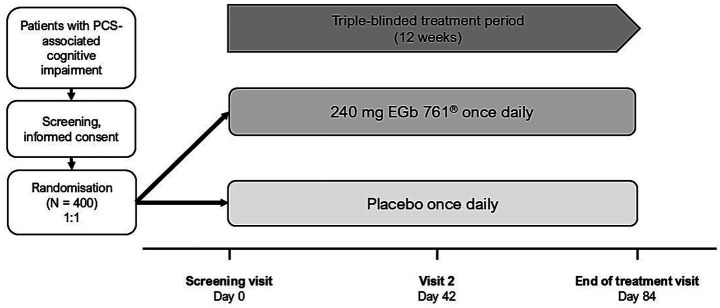
Trial flow chart.

Prospective participants will undergo a screening process prior to administration of the intervention (Figure 1). All participants who enter screening for the trial will receive a unique 5-digit participant identification number assigned by the IRT system, consisting of the 1-digit country code, the 2-digit centre code, and the 2-digit participant chronological number. If eligible for enrolment, the participant number will be associated with a medication number via the IRT system. Investigators, all site and sponsor personnel are unaware of the randomisation list.

Randomisation will be carried out by clinical research organisation. Medication numbers will be generated from 5,001 onwards (specified within a randomisation specification and authorisation form) and are allocated proportionally to the treatment arms in a 1:1 ratio. The intervention will be provided, packed, and labelled with the medication number in accordance with randomisation. The intervention will be dispensed using IRT. At the baseline visit (Visit 1, Day 0), after baseline assessments are completed, the investigator will log in to the interactive response technology system to dispense the medication number allocated to the patient.

### 2.5 Treatment

Placebo was chosen as comparator to facilitate blinding of treatment, as the gold standard in randomised controlled trials. Furthermore, there is no commonly accepted therapy available for the treatment of cognitive impairment associated with PCS which precludes the use of an active comparator.

Symptoms in PCS have been observed to fluctuate (Burton et al., 2023), to improve without treatment (Troscher et al., 2024), and to be susceptible to expectations (Matta et al., 2022; Salzmann et al., 2024). Relevant improvements with placebo treatment have been reported in this population (Geng et al., 2024; Redel et al., 2024). Practise effects are inherent to repeated cognitive testing (Bell et al., 2018). A clear differentiation between any such unspecific effects and pharmacologic effects can only be achieved by a randomised, placebo-controlled, blinded trial. According to the declaration of Helsinki, a placebo is justified in situations where no proven intervention exists and the patients who receive placebo will not be subject to additional risks of serious or irreversible harm (World Medical Association, 2024). This is exactly the situation in PCS and therefore the use of placebo is justified.

Patients in the EGb 761® arm will receive one film-coated tablet containing 240 mg EGb 761® (*Ginkgo biloba* extract) once daily. Being an approved herbal medicinal product in many EU countries, EGb 761® is manufactured according to Good Manufacturing Practice under strict quality management and regulatory inspections. Complete quality control of multiple parameters over the whole production process ensure that each batch complies with the requirements of the European Pharmacopoeia (e.g., contains 22.0–27.0% ginkgo flavonoids calculated as ginkgo flavone glycosides and 5.4–6.6% terpene lactones consisting of 2.8–3.4% ginkgolides A, B, C, and 2.6–3.2% bilobalide and less than 5 ppm ginkgolic acids) (Kulić et al., 2022).

Patients in the placebo arm will receive a matching placebo tablet once daily. The tablets are to be swallowed whole with a sufficient amount of water (about 200 mL). The placebo tablet has a similar appearance to EGb 761® tablets, with matching coating and core, but does not contain any active components. The film-coating prevents unmasking by the distinctive taste of *Ginkgo biloba* extract. Tablets are to be taken orally each morning for 12 weeks. Dose modification is not allowed. As participants will self-administer treatment at home, compliance with the trial treatment will be assessed at each study visit by direct questioning and counting returned tablets. Deviations from the dosage regimen will be recorded in the electronic case report form (eCRF). A record of the number of tablets dispensed to and taken by each participant must be maintained and reconciled with the treatment and compliance records. Start and stop dates of trial treatment, including dates for delays, will also be recorded.

Patients who wish to continue taking the study drug after the final visit may be able to purchase it from pharmacies.

### 2.6 Outcomes

#### 2.6.1 Efficacy outcomes

As no scientific standard has been developed to date^1^, a wide range of cognitive, neuropsychiatric, neurosensory, global, and functional outcomes have been included in this trial to evaluate the effect of EGb 761® (240 mg once daily) compared to placebo (Table 2). These outcomes cover the most salient cognitive deficits in PCS and also the main symptoms of the disease, especially those linked to cognitive symptoms (e.g., fatigue, neuropsychiatric symptoms).

**Table 2:** Clinical objectives, trial assessments, and timeframe.

Clinical objectives	Assessments and timeframe
To evaluate the effect of EGb 761® on objective cognitive outcomes in participants with cognitive impairment associated with PCS	Mean change from baseline to Week 6 and Week 12 in the following test scores: Digit Span Forward and Backward test (Wechsler, 1997) Verbal Fluency test (Isaacs and Kennie, 1973) TMT (A + B) (Tombaugh, 2004) CVLT-II (Delis et al., 2000) d2-R (Brickenkamp et al., 2015)
To evaluate the effect of EGb 761® compared to placebo on neuropsychiatric outcomes in participants with cognitive impairment associated with PCS	Mean change from baseline to Week 6 and Week 12 in the following questionnaire scores: GAD-7 (Spitzer et al., 2006) PHQ-9 (Kroenke et al., 2001)
To evaluate the effect of EGb 761® compared to placebo on neurosensory outcomes in participants with cognitive impairment associated with PCS	Mean change from baseline to Week 6 and Week 12 in the 11-Point Box Scale score for vertigo and tinnitus (Spiegel et al., 2018)
To evaluate the effect of EGb 761® compared to placebo on global and functional outcomes in participants with cognitive impairment associated with PCS	Mean change from baseline to Week 6 and Week 12 in the following test/questionnaire scores: CGI-S (Busner and Targum, 2007) PCFSS (Klok et al., 2020) MFIS (Strober et al., 2020) mPEM-DSQ (Froehlich et al., 2021) SBQ-LC (Hughes et al., 2022) CGI-I^a^ (Busner and Targum, 2007)

CGI-I, Clinical Global Impressions-Improvement; CGI-S, Clinical Global Impressions-Severity; CVLT, California Verbal Learning Test; d2-R, d2 Test of Attention-Revised; GAD-7, Generalised anxiety disorder-7 item; PCFSS, Post-COVID-19 Functional Status scale; PCS, Post COVID-19 syndrome; PHQ-9, Patient Health Questionnaire-9; MFIS, Modified Fatigue Impact Scale; mPEM-DSQ, Modified Post–Exertional Malaise Subscale questions from DePaul Symptom Questionnaire; SBQ-LC, Symptom Burden Questionnaire for Long COVID; TMT (A + B), Trail-Making Test Parts A and B.

a CGI-I will be assessed at Week 6 and Week 12 only, not at baseline.

#### 2.6.2 Safety outcomes

Safety assessments will include the nature and frequency of adverse events (AEs), physical examinations, and vital signs. The number of participants with AEs (regardless of causality and seriousness), adverse drug reactions, and serious AEs will be summarised by treatment arms and exposure phase. AEs will be coded using MedDRA to classify events under primary system organ class and preferred term.

A complete physical examination will be performed at baseline (Visit 1, Day 0) and will include, at a minimum, assessments of the cardiovascular, respiratory, gastrointestinal, and neurologic systems. Physical examination will be repeated at the end of treatment (Visit 3, Day 84–98). Vital signs, including pulse rate, respiratory rate, and blood pressure, will also be assessed at baseline and end of treatment.

Any monitoring activities based on concomitant diseases or treatments will be continued.

### 2.7 Study schedule

Participants will start treatment on Day 1 and will self-administer the investigational product once daily at home for 12 weeks (Figure 1). During the treatment period, participants will visit the trial sites twice, in Week 6 (Visit 2, Day 42 ± 3) and Week 12 (Visit 3, Day 84 + 14). End-of-trial assessments will be performed on the last trial visit (Visit 3, Day 84 + 14) or prior in case of withdrawal from the trial.

### 2.8 Sample size calculation

Currently, there is very limited data on the use of EGb 761® in patients with cognitive impairment associated with PCS (Gebetsroither, 2024; Zifko et al., 2022). Therefore, the sample size was determined considering a small standardised mean difference (treatment effect size) according to Cohen’s d (Cohen, 1988).

A treatment effect size was reported as 0.52 for EGb 761® compared to placebo for cognition in a meta-analysis of dementia trials (Gauthier and Schlaefke, 2014). Because patients with dementia are more cognitively impaired and represent a more homogeneous population compared to patients with PCS, the treatment effect size is assumed to be smaller in PCS. To detect at least one significant outcome in the two cognitive domains memory and executive functioning assuming a treatment effect size of at least 0.25, a correlation of 0.3 to 0.4 between both domains (Napryeyenko et al., 2007; Ihl et al., 2011; Herrschaft et al., 2012; Nikolova et al., 2013), a statistical power of at least 80%, a local 2-sided significance level of 0.05, and a dropout rate of about 15%, 400 patients need to be randomised (R package rPowerSampleSize Version 1.0.2, R version 4.3–2 [2023-03-15]).

### 2.9 Data collection and management

#### 2.9.1 Data collection

Multiple tests and questionnaires assessing cognitive, neuropsychiatric, neurosensory, global, and functional outcomes will be administered throughout the trial (schedule described in Table 3). These include the California Verbal Learning Test (CVLT), Digit Span Forward and Backward test (Wechsler, 1997), d2 Test of Attention – Revised (d2-R) (Brickenkamp et al., 2015), Trail-Making Test (TMT) Parts A and B (Tombaugh, 2004), Verbal Fluency Test (Isaacs and Kennie, 1973), Generalised Anxiety Disorder-7 item (Spitzer et al., 2006), Patient Health Questionnaire-9 item (Kroenke et al., 2001), 11-Point Box Scale score for vertigo and tinnitus (Spiegel et al., 2018), Clinical Global Impressions – Severity (Busner and Targum, 2007), Post-COVID-19 Functional Status scale (Klok et al., 2020), Modified Fatigue Impact Scale (Strober et al., 2020), Modified Post-Exertional Malaise Subscale questions from DePaul Symptom Questionnaire (Froehlich et al., 2021), Symptom Burden Questionnaire for Long COVID (Hughes et al., 2022), and Clinical Global Impressions – Improvement (CGI-I) (Busner and Targum, 2007).

**Table 3:** Study schedule.

Period	Baseline	Treatment	
Visit	1	2 (After 6 weeks ±3 days)	3 (After 12 weeks + 14 days)
Trial entry and general assessments			
Informed consent	X		
Inclusion/exclusion criteria	X		
Demographics	X		
Medical/surgical history	X		
Prior and concomitant medications and procedures	X	X	X
Physical examination	X		X
Vital signs	X		X
Pregnancy stick test	X		X
AE surveillance		X	X
Randomisation	X		
Investigational product			
Dispensing	X	X	
Return and accountability		X	X
Cognitive testing			
CVLT – learning, immediate and short delay trials	X	X	X
Digit span forward and backward	X	X	X
TMT (A + B)	X	X	X
CVLT long delay trials	X	X	X
Verbal fluency test	X	X	X
d2-R	X	X	X
Clinician rating			
PCFSS	X	X	X
CGI-S	X	X	X
CGI-I		X	X
Participant self-rating scales			
11-Point Box Scales for Vertigo	X	X	X
11-Point Box Scales for Tinnitus	X	X	X
GAD-7	X	X	X
PHQ-9	X	X	X
PCFSS	X	X	X
MFIS	X	X	X
SBQ-LC	X	X	X
mPEM-DSQ	X	X	X

To ensure the standardised conduction of the cognitive tests and to improve the reliability of the ratings, all investigators and raters will be intensively trained on all cognitive and clinician-rated tests applied in this clinical trial. The training will be performed by a recognised expert in cognitive testing during the investigator meeting or a separate rater training prior to the site initiation visit.

#### 2.9.2 Data management

Data entry will be carried out using a validated electronic remote data capture system which complies with Food and Drug Administration (FDA) regulation 21 Code of Federal Regulation Part 11. Clinical data management will be performed in accordance with applicable GCP standards and data cleaning procedures to ensure the integrity of the data. All eCRF entries, corrections, and alterations must be made by the investigator or authorised site staff. Discrepancies in the data will be brought to the attention of the clinical team, and site staff, if necessary. Resolutions to these issues will be reflected in the database and an audit trail within the system will track all changes made to the data.

### 2.10 Follow-up, study termination and withdrawal

All adverse events (AEs) or serious AEs reported or observed during the trial will be recorded on the eCRF. AEs resulting from concurrent illnesses, reactions to concurrent illnesses, reactions to concurrent medications, or progression of disease states must also be reported. All AEs will be followed to adequate resolution. All participants with AEs potentially related to the investigational products that are unresolved at the end of trial (or upon early withdrawal) must be followed up. The investigator will follow up with all participants experiencing AEs until resolution or stabilisation the event.

Participants may discontinue treatment or withdraw consent at any time for any reason, or if they withdraw consent due to not tolerating the trial treatment, not perceiving a benefit from the trial treatment, or preferring an alternative treatment. They may also discontinue from the trial if they experience a serious AE that in the investigator’s opinion require discontinuation from the trial, or develop a new medical condition that renders trial participation unsafe. Reasons for discontinuing trial treatment will be recorded.

### 2.11 Statistical methods

The efficacy endpoints will be summarised using summary statistics in line with estimand strategies. Intercurrent events considered in this trial are treatment discontinuation, treatment noncompliance and initiation or adjustment of prohibited concomitant medication. The hypothetical strategy will be used to estimate the effects attributable to trial treatment without influence from prohibited medication and where the treatment is taken as intended. For the safety endpoints, the incidence of participants with AEs, adverse drug reactions, and serious AEs for the safety population will be summarised by treatment arms and exposure phases. The analysis will be exploratory in nature, and no formal hypothesis testing for confirmatory claims will be performed. Therefore, no primary endpoint has been selected, endpoints are not hierarchically ordered or weighted in the analysis and no adjustments for multiplicity will be applied. However, descriptive *p*-values will be provided with appropriate tests.

The full analysis set will be used for the efficacy analyses and the safety set for the safety analyses. Predefined subgroup analyses will be conducted for gender and age groups. Data collected in this trial will be presented using summary tables, patient data listings, and figures. Models such as the analysis of covariance (ANCOVA) and a mixed-effects model for repeated measures (MMRM) will also be explored to compare the efficacy parameters between the treatment groups. For the ANCOVA model, treatment will be included as factor, and baseline value will be included as continuous covariate. The MMRM will incorporate the Huber-White “Sandwich” standard error and will include visit, treatment, visit by treatment as factors, and baseline value (baseline value for the corresponding endpoint analysed) as a continuous covariate. Summary statistics will be provided for demographics, medical/surgical history, and physical examination at baseline and prior/concomitant medication. The homogeneity of treatment groups will be assessed through analysis of baseline characteristics. Additional details of all analyses and data reviews will be provided in the statistical analysis plan (SAP). A draught SAP will be developed prior to the randomisation of the first participant and will be finalised and signed off before the database is locked.

For categorical and ordinal variables, percentages will be calculated based on the number of participants in the corresponding analysis set and the number of participants with missing data will also be included. Additional details of handling of missing data or data not used after the occurrence of an intercurrent event by multiple imputation methods will be provided in the SAP.

Statistical analysis will be performed by an independent external service provider (Thermo Fisher Scientific, Wilmington, United States).

## 3 Discussion

EGb COCOS will be performed to determine the suitability of EGb 761® as a treatment for cognitive impairment associated with PCS in a randomised, placebo-controlled trial.

The cognitive impairment observed in PCS is thought to be driven by a complex and multifactorial pathophysiology, including persistent neuroinflammation, oxidative stress, mitochondrial dysfunction, and vascular and endothelial dysfunction (Chen et al., 2025; Mueller and Müller, 2024; Peluso and Deeks, 2024). EGb 761® is a multi-target agent whose broad pharmacological spectrum provides a consistent mechanistic rationale for its use by addressing these pathological pillars (Mueller and Müller, 2024; Owens et al., 2024). Specifically, EGb 761® exhibits potent anti-inflammatory properties that counter the microglial activation and cytokine release (e.g., IL-6) (Sun et al., 2024), which have been postulated to play a central role in post-COVID-19 neuroinflammation (Mueller and Müller, 2024; Peluso and Deeks, 2024). EGb 761® has also been shown to exhibit significant antioxidant activity, mitigating oxidative stress and mitochondrial dysfunction (Eckert et al., 2005). Furthermore, EGb 761® has beneficial effects on the vascular system, including the protection of endothelial cells and reduction of blood viscosity (Zhang et al., 2017; ErdinҪler et al., 1996). Taken together, these properties of EGb 761® could directly address the impairment of neurovascular coupling (Owens et al., 2024). Neurovascular coupling refers to the coordinated interplay between brain parenchymal cells and vascular cells that constitute the neurovascular unit. Under normal conditions, it adjusts the cerebromicrovascular blood flow to meet the neuronal demands on energy and nutrients. Neurovascular uncoupling has been proposed as a critical component of cognitive dysfunction in PCS (Owens et al., 2024). Finally, EGb 761® enhances neuroplasticity and promotes neuroprotection, in part by increasing the levels of brain-derived neurotrophic factor (BDNF) (Xu et al., 2007; Akanchise and Angelova, 2023), which is known to be diminished in PCS (Chen et al., 2025). Through these pleiotropic actions, EGb 761® may be a mechanistically plausible and low-risk therapeutic option for PCS-associated cognitive impairment (Owens et al., 2024; Zifko et al., 2022).

Whilst most placebo-controlled trials supporting the efficacy of EGb 761® in mild cognitive impairment and dementia used treatment durations of 22 to 52 weeks, clinical benefits on cognition have been reported with 6 to 12 weeks of treatment in healthy individuals and patients with very mild cognitive impairment (Kaschel, 2009; Grass-Kapanke et al., 2011; Kaschel, 2011; Beck et al., 2016). Cognitive impairment in PCS has been reported to improve spontaneously within 6 to 12 months in a majority of patients. Therefore we concluded that a clinical effect not emerging within 3 months of treatment would be of limited clinical benefit in this population and selected a 12 week treatment schedule.

Strengths of the present trial include its large sample size, randomisation, triple-blinding, and placebo-comparator arm. According to the WHO, PCS occurs in individuals with a history of probable or confirmed SARS-CoV-2 infection continuing at least 3 months from the onset of COVID-19, with symptoms lasting at least 2 months, that cannot be explained by an alternative diagnosis (World Health Organization, 2021). By using this definition in the inclusion criteria, this clinical trial aims to include patients with a documented history of probable or confirmed SARS-CoV-2 infection. The findings of the trial may prove highly valuable for patients with cognitive impairment associated with PCS. In addition, the methodology implemented in the study may be useful for future trials addressing cognitive issues in PCS.

The trial outcomes and assessments are easy to record, location-independent, and applicable across a diverse patient population. A narrative review of pharmacological studies evaluating treatment options for neuropsychiatric symptoms in PCS shows the current wide range of cognitive endpoints used (Lejay et al., 2023). No standardised approach has yet been established (see text Footnote 1). Notably, there is accumulating evidence of a discrepancy between subjective cognitive complaints and objective cognitive impairment in patients with PCS (Bland et al., 2024). Subjective complaints are more likely to be reported than objectively detected (Tarantini et al., 2025; Schild et al., 2023), highlighting the limitations of cognitive testing in this patient population. In the present trial, an exploratory trial design will be used that includes a range of validated cognitive endpoints (e.g., Digit span Forward and Backward, Verbal Fluency test, Symptom Burden Questionnaire for long COVID) (SBQ-LC, module “Memory, Thinking and Communication”). These have been chosen to cover both general cognitive impairment and COVID-19-associated cognitive impairment. Additionally, other symptoms linked to cognitive complaints and mediating the relationship between subjective cognitive symptoms and objective cognitive performance will be examined (Delgado-Alonso et al., 2025a; Denno et al., 2025). Post-exertional malaise (PEM) is a key feature of PCS and an important consideration for trial participants. One measure to limit the PEM risk was to restrict cognitive testing to the minimum required to achieve the trial objective. Therefore we restricted cognitive testing to those domains that have been reported to be affected most in PCS and to be most relevant for everyday life in PCS (Delgado-Alonso et al., 2022, Delgado-Alonso et al. 2025a, Panagea et al., 2025). Validated tests with available versions in Spanish, Polish, and German were selected for working memory (Digit Span Forward and Backward), verbal learning (California Verbal Learning Test), executive function (Trail-Making Test), verbal fluency, and sustained attention (d2 Test of Attention – Revised). Applying these tests as a fixed battery will allow to assess fatigability, a key feature of cognitive complaints in PCS that is poorly captured by other validated cognitive tests. Whilst this approach might miss treatment effects in cognitive domains not tested, such as visuospatial abilities or social cognition, the cognitive battery covers the domains most relevant in PCS.

Consultation on the trial design and planned tests and procedures was requested from the German patient organisation “Long COVID Deutschland” (LCD). Two measurements have been implemented in the trial design, based on their recommendation. Firstly, the electronic case report form now includes a section for the recording of any self-medication and the use of food or dietary supplements, vitamins, and minerals due to the widespread use of such products amongst patients with PCS. Secondly, the visits to the trial sites will be standardised to reduce avoidable burden on participants.

EGb 761® is generally well tolerated. In clinical trials, adverse events have not been reported more frequently than with placebo (Tan et al., 2015). Known contraindications and warnings (e.g., pregnancy, hypersensitivity to the investigational product, haemorrhagic diatheses, coagulation disorder, concomitant use of anticoagulants) are exclusion criteria for the trial.

Symptoms of depression and anxiety are frequent in PCS (Bidhendi-Yarandi et al., 2025; Seighali et al., 2024). In samples from the general population, both depression and anxiety are associated with impairments in cognitive function, e.g., (Lindert et al., 2021; Suddell et al., 2023). By contrast, in a large PCS cohort, mild to moderate depression had only a limited impact on objective cognitive performance (Delgado-Alonso et al., 2025a; Delgado-Alonso et al., 2022; Delgado-Alonso et al., 2025b). The interpretation was that in PCS fatigue and other factors have stronger effects on cognition than anxiety and depression. In PCS, fatigue and depression are highly correlated and can be difficult to distinguish (Stallmach et al., 2022). Considering that fatigue is a cardinal feature of PCS, and that depression and anxiety are very frequent in PCS, we decided to exclude only patients with severe depression or anxiety. Excluding patients with (treated) mild-to-moderate anxiety and depression would have excluded a large part of the PCS population. The vast majority of patients seeking medical care for PCS meets our liberal inclusion criteria of at least mild depression (PHQ-9 score> = 5) or anxiety (GAD-score> = 5) (Stallmach et al., 2022; Downer et al., 2024). Our main strategy to mitigate potential confounding by concomitant mild-to-moderate depression and anxiety as well as their treatments is trial design. Potential effects of known and unknown confounders on outcomes will occur in both treatment groups in a randomised parallel group trial. In case treatment groups will be unbalanced in important known potential confounders despite randomization, statistical analysis will account for such differences.

Patients that were on mechanical ventilation in an ICU during acute SARS-CoV-2 infection are excluded because we want to avoid confounding with post intensive care syndrome (PICS). Because PCS and PICS might differ in pathophysiology (Fleischmann-Struzek et al., 2024) and PCS also occurs after non-severe acute COVID-19, excluding ICU/mechanical ventilation results in a more post-COVID specific sample.

Cognitive performance typically declines in middle-aged and elderly people in the general population. In post-COVID syndrome (PCS) however, subjective and objective cognitive impairment has been reported over all adult age groups (Gonzalez Aleman et al., 2025). Whilst age is a risk factor for cognitive impairment in PCS, cognitive impairment might have a more profound effect on daily life in younger cohorts (Davis et al., 2021). Therefore treatment of cognitive impairment in PCS is clinically relevant over all age groups and we include adult patients without further age restriction. Because age-specific phenotypes have been reported for cognitive impairment in PCS (Gonzalez Aleman et al., 2025), we plan a subgroup-analysis by age.

The trial design has several limitations. First, to limit the burden to patients and the duration of trial visits, a decision was taken not to conduct imaging studies and not to collect biological samples. As a consequence, we will be able to assess clinical effectiveness, but we will be unable to explore potential modes of action. Second, for the reasons discussed above the statistical analysis will be exploratory in nature, and no formal hypothesis testing for confirmatory claims will be performed. With an observation time of 12 weeks we will not be able to evaluate long-term effects. Excluding patients with a history of prolonged PEM excludes ME/CFS-like phenotypes and therefore limits generalizability of results. In case we will observe efficacy and good tolerability in the present trial, these limitations need to be addressed in future studies.

## Footnotes

1 Mey, D. (2023) Inventory of Cognitive Tests to Characterise Brainfog in Long-/Post-Covid.

## Glossary

CVLT: California verbal learning test
d2-R: d2 test of attention-revised
eCRF: electronic case report form
GAD-7: generalised anxiety disorder 7-item
GCP: good clinical practise
ICH: international council for harmonisation
IEC: independent ethics committee
IRT: interactive response technology
MedDRA: medical dictionary for regulatory activities
MFIS: modified fatigue impact scale
mPEM-DSQ: modified post-exertional malaise subscale questions from DePaul symptom questionnaire
PCFSS: post COVID functional status scale
PCS: post COVID-19 syndrome
PCR: polymerase chain reaction
PEM: post-exertional malaise
PHQ-9: patient health questionnaire-9
SAP: statistical analysis plan
SARS-CoV-2: severe acute respiratory syndrome coronavirus 2
SBQ-LC: symptom burden questionnaire for long COVID
TMT (A + B): trail-making test parts A and B
